# Designing programs to improve diets for maternal and child health: estimating costs and potential dietary impacts of nutrition-sensitive programs in Ethiopia, Nigeria, and India

**DOI:** 10.1093/heapol/czy013

**Published:** 2018-03-07

**Authors:** William A Masters, Katherine L Rosettie, Sarah Kranz, Goodarz Danaei, Patrick Webb, Dariush Mozaffarian, the Global Nutrition and Policy Consortium, Lalita Bhattacharjee, S Chandrasekhar, Cheryl Christensen, Sonalde Desai, Nabeeha Kazi-Hutchins, Carol Levin, Robert Paarlberg, Steven Vosti, Olayinka Adekugbe, Gudina Egata Atomsa, Jane Badham, Kaleab Baye, Mesfin Beyero, Namukolo Covic, Babukiika Dalton, Charlotte Dufour, Patrizia Fracassi, Zewditu Getahun, Jemal Haidar, Tesfaye Hailu, Aweke Kebede, Joyce Kinabo, Jamal Bakari Kussaga, George Mavrotas, Wilson Waiswa Mwanja, Babatunde Oguntona, Abiodun Oladipo, Ruth Oniang’o, Simbarashe Sibanda, Roger Sodjinou, Carol Tom, Henry Wamani, Akwilina Wendelin, Ramesh Kant Adhikari, Archana Amatya, Manav Bhattarai, Viral Brahmbhatt, Ram Krishna Chandyo, Seema Gulati, Umesh Kapil, Ranju Mehta, Sailesh Mohan, D Prabhakaran, V Prakash, Seema Puri, S K Roy, Rekha Sharma, Sabnam Shivakoti, Andrew Thorne-Lyman, Pooja Pandey Rana, Geeta Trilok-Kumar

**Affiliations:** 1 Friedman School of Nutrition Science and Policy, Tufts University, 150 Harrison Ave, Boston, MA 02111, USA; 2 Department of Global Health, T.H. Chan School of Public Health, Harvard University, 677 Huntington Ave, Boston, MA 02115, USA

**Keywords:** Food, nutrition, agriculture, policy evaluation, child health

## Abstract

Improving maternal and child nutrition in resource-poor settings requires effective use of limited resources, but priority-setting is constrained by limited information about program costs and impacts, especially for interventions designed to improve diet quality. This study utilized a mixed methods approach to identify, describe and estimate the potential costs and impacts on child dietary intake of 12 nutrition-sensitive programs in Ethiopia, Nigeria and India. These potential interventions included conditional livestock and cash transfers, media and education, complementary food processing and sales, household production and food pricing programs. Components and costs of each program were identified through a novel participatory process of expert regional consultation followed by validation and calibration from literature searches and comparison with actual budgets. Impacts on child diets were determined by estimating of the magnitude of economic mechanisms for dietary change, comprehensive reviews of evaluations and effectiveness for similar programs, and demographic data on each country. Across the 12 programs, total cost per child reached (net present value, purchasing power parity adjusted) ranged very widely: from 0.58 to 2650 USD/year among five programs in Ethiopia; 2.62 to 1919 USD/year among four programs in Nigeria; and 27 to 586 USD/year among three programs in India. When impacts were assessed, the largest dietary improvements were for iron and zinc intakes from a complementary food production program in Ethiopia (increases of 17.7 mg iron/child/day and 7.4 mg zinc/child/day), vitamin A intake from a household animal and horticulture production program in Nigeria (335 RAE/child/day), and animal protein intake from a complementary food processing program in Nigeria (20.0 g/child/day). These results add substantial value to the limited literature on the costs and dietary impacts of nutrition-sensitive interventions targeting children in resource-limited settings, informing policy discussions and serving as critical inputs to future cost-effectiveness analyses focusing on disease outcomes.

Key Messages

- Existing evidence on cost-effectiveness for nutrition improvement focuses on interventions to address specific diseases. We provide a novel participatory approach to assembling cost and impact data for 12 nutrition-sensitive interventions to improve diet quality in three countries: Ethiopia, Nigeria and India. Programs designed by stakeholders often use resource transfers to influence diets despite their high cost; programs altering food access have lower cost. Future work using these data will analyse net cost-effectiveness.

## Background and motivation

Undernutrition among children in low-income settings is among the world’s leading causes of death, disability and inequity (Black *et al.* 2008; GBD 2016 Risk Factors Collaborators 2017). Governments in low- and middle-income countries around the world increasingly acknowledge child nutrition as a high priority, with specific targets for improvements by 2025 (United Nations 2016). National governments and international agencies declared a ‘Decade of Action for Nutrition’ starting in 2016 (Food and Agriculture Organization of the United Nations and World Health Organization 2016).

To achieve these goals, novel programs are needed that address overall dietary diversity and quality (Haddad *et al.* 2016). Yet, most available evidence to-date focuses on nutrient supplementation (Bhutta *et al.* 2013), with far less evidence on relative costs and effectiveness of programs that aim to improve dietary quality through nutrition-sensitive actions such as changes in home production, education or purchasing power (Ruel *et al.* 2013). Nutrition-sensitive interventions can be defined as strategies that address underlying causes of insufficient or inadequate food such as poor agricultural production, limited food markets, low levels of education or weak purchasing power. Nutrition-sensitive programs frequently involve multiple sectors and more diverse stakeholders than supplementation programs, requiring different kinds of evidence and priority-setting processes (Development Initiatives 2017). While many such programs are now being designed and implemented to improve diet quality in low-income countries (Hoddinott *et al.* 2013), scare empirical evidence exists on their costs and on their impacts on dietary intake.

The purpose of this study is to fill evidence gaps about the costs and impacts of nutrition-sensitive interventions that could potentially be implemented to improve child nutrition in sub-Saharan Africa (SSA) and South Asia. Through consultation with regional experts, we identified the types of interventions likely to be of greatest interest to development actors, delineated the mechanisms and magnitudes by which those actions might alter diets, compared expert consensus views to previously estimated costs and impacts of similar programs undertaken at other times and places, and summarized the implications of this process for priority-setting. Strengths of this approach include its independence from the interests of parties involved in such interventions, which can introduce bias when analyses of program costs and impacts are undertaken by the implementing agency or program funder; and its participatory nature, drawing on local expertise and incorporating perspectives of diverse stakeholders to maximize regional generalizability and relevance.

## Methods

This study estimated costs and impacts on dietary intake of priority nutrition-sensitive programs to improve maternal-child health in SSA and South Asia. Our mixed methods approach included regional meetings with expert stakeholders from a variety of institutional, sectoral and disciplinary backgrounds in SSA and South Asia; delineation of program components and economic mechanisms for dietary change; and literature reviews to validate and calibrate estimated program costs and impacts on dietary intake. Additional details on these processes are outlined below, and the analytical framework is described in Table 1.

**Table 1.** Analytical framework for estimating program costs and impacts

Costing framework	
Component of costing framework	Description
Cost category and item description	Items are grouped into program cost categories, including: personnel (by level of salary range); real estate for office space and other needs; transportation costs; supplies, equipment, other resources; monitoring and evaluation as a percent of other program costs; and other costs or revenue. Separate lines within each category are used for individual items with differing prices or numbers of units
Units of measure	Units of measure are explicitly listed, such as person-years for salaries, kilometres travelled for transport, and workshop days for attendee expenses
Price per unit	Price per unit is calculated by converting local currency amounts to constant US dollars in PPP terms, so that costs are comparable across countries and over time
Start-up costs	Calculated using the number of units and cost per unit (quantity×price) during the first year of the program
Recurring costs	Calculated using the number of units and cost per unit (price×quantity) for each year after the first, using a standard inflation rate in PPP prices of 0.05
NPV	Calculated as the sum of all items across duration of each program, with a discount rate over time of 0.03


Dietary impact framework^a^			
Mechanism for impact	Description of impact mechanism	Main program parameters	Main behavioural parameter
Resource transfers	Transfer of resources to shift composition of diet	Number of targeted individuals, and value of resource transfer to them, as a percent of their total income	Income elasticity of demand for the targeted food item
Access changes	Changing food prices to alter purchasing behaviour	Number of consumers affected, and percent change in their cost of acquisition of the targeted food item	Price elasticity of demand for the targeted food item
Preference change	Changing dietary preferences	Number of consumers affected by the program’s behaviour-change efforts	Change in quantity of nutrient consumed per recipient per day
Food transfers	Transfer of food items to increase intake of target nutrients	Number of recipients to whom food is transferred	Change in quantity of nutrient consumed per recipient per day


PPP, purchasing power parity

a Each program may aim to alter intake of more than one food, through more than one mechanism of impact as described by program parameters that describe its reach and delivery, and the resulting alteration of dietary intake depends on behavioural parameters obtained from the best available studies of similar changes in similar contexts, as specified in Table 5

### Selection of programs aiming to improve diet quality

To identify a set of programs most likely to be high priorities for government or donor funding, we organized and held in-person meetings with a range of regional nutrition and program experts on South Asia (hosted in Nepal in December 2015) and SSA (hosted in Ethiopia in February 2016). The goal of these meetings was to identify nutrition-sensitive programs that local experts consider to be of greatest relevance to child nutrition in eight countries with high burdens of undernutrition: India, Nepal, Bangladesh, Ethiopia, Nigeria, Ghana, Tanzania and Uganda. For this analysis we retained the 12 programs for which a full set of cost and impact data could be calculated, which limits coverage to India, Ethiopia and Nigeria.

Our participatory approach ensured that interested parties could not pre-determine which programs would be considered or how their cost-effectiveness would be calculated. At these meetings, a total of 48 specific nutrition-sensitive programs were considered, identified based on interventions that were currently being implemented, under debate as potential additions to existing activities, or new programs with high promise for efficacy. For each proposed program, the following information was discussed: (1) the description of the program; (2) the mechanisms for impact on dietary behaviours; (3) the target foods and nutrients to be increased; (4) the location and demographic characteristics of the target population; (5) the lead authority and implementing organization for the program; (6) the types and costs of resources required for program implementation, using an ingredients approach (unit needs and costs) and separately considering start-up, recurring costs and evaluation; and (7) the additional regional expert contacts relevant to that program. Additional details on the methods and results of these two regional meetings are documented elsewhere (Masters *et al.* 2017).

From the 48 programs identified at our regional expert meetings, we focused on 12 for analysis in this paper (Table 2) based on the following three criteria: First, we included only programs that participants described as relevant for India, Ethiopia or Nigeria, or for the South Asian or African contexts more generally, so as to align results with country priorities of the Bill & Melinda Gates Foundation which supported this project. Next, we included only programs that targeted children under 5, relevant to linking changes in dietary intake to disease outcomes for maternal-child health. Finally, we excluded programs for which required resources for implementation were not sufficiently documented to compute program costs.

**Table 2.** Program elements by country

Country	Program name	Program description^a^	Target population^b^^c^	Dietary risk factor targeted^d^
Ethiopia	Conditional livestock transfer	Provides one dairy cow per target household, conditional on pregnant mother’s ANC attendance	Children under 5 in the PSNP with pregnant mothers	Cow’s milk, zinc, vitamin A, animal protein
	Conditional poultry transfer	Provides 2 hens and 1 cock to recipient households conditional on men engaging in public works programs and women/children attending ANC/child vaccination and health visits	Children under 5 in the PSNP	Eggs, **vitamin A, animal protein, zinc, iron**
	Media & education campaign	A radio and education campaign that focuses on increasing intake of animal and plant-based protein, as well as meal frequency using radio segments nutrition messages delivered by religious leaders.	Children under 5 living in rural areas	Meat, milk, eggs, fish, plant protein sources, **iron**
	Educational entertainment	Peer-to-peer videos delivering nutrition messages, coupled with community discussions of prenatal nutrition	National children under 5	Eggs, **vitamin A, animal protein, zinc, iron**
	Complementary food production	Education to women on how to wash, dry, mill, and fortify grains with a micronutrient powder to produce complementary foods for their own use or to sell	Children under 5 living in semi-urban areas	Grains, maize, sorghum, teff, wheat, barley, pulses, legumes, **zinc, iron**
Nigeria	Conditional cash transfer	Cash transfers to pregnant women conditional on ANC attendance by mother and family member, and delivery in health facility	Children under 5 living in rural areas with pregnant mothers	**Iron**
	Food pricing program	A flat 10% tax on SSBs to fund FV subsidies for mothers and children	National children under 5	Fruits, vegetables, **vitamin A**
	Complementary food processing and sales	Teaching women to produce and sell affordable cereal-based CF mixed with powdered pulses and dried animal-based foods; coupled with nutrition education on complementary food	Children 6–24 months in two low-income regions of Nigeria	Cereals, pulses, soy, fish, chicken, lentils, cowpeas, **zinc, iron, animal protein, vitamin A**
	Household animal & horticulture production	Provides seedlings, seeds, and chickens, as well as training on food and poultry production, to targeted households with an able body and plot of land with	Children under 5 living in households in the poorest 40% of population	Fruits, vegetables, chicken, **zinc, iron, animal protein, vitamin A**
India	Complementary food processing	Provides a monthly ration of locally produced micronutrient sachets, coupled with education on how to add the sachets to complementary food	Children 6–24 months in poorest 50% of population	**Zinc, iron**
	Diet diversity media campaign	Mass media radio campaign focusing on raising consumption of vitamin A-rich foods; coupled with community cooking demonstrations	Children under 5 living in one district	Carrots, pumpkin, mango, **vitamin A**
	Home gardens	Establishes home gardens for households with agricultural or homestead land; provides seeds, supplies, and tools; coupled with education and resources for small livestock/poultry production	Children under 5 living in rural households	Yellow/orange vegetables, dark green leafy vegetables, animal source foods, **zinc, vitamin A, iron, animal protein**

PSNP, productive safety net program; ANC, antenatal care; ND, no data; SSB, sugar-sweetened beverage; FV, fruit and vegetable

a Program descriptions are based on consensus formed by stakeholders at regional meetings in Nepal and Ethiopia

b The target population is the population that each program’s impact will be assessed in. Impact estimates are restricted to children under 5 to complement the current version of the model

c In cases where regional experts did not specify the target population size, regional data sources such as census data, Demographic Health Surveys, and UN Population Division estimates, were used to approximate target population sizes

d Targeted risk factors may be foods, or specific nutrients within foods (in bold text)

### Determination of program impacts

To estimate the impacts of each intervention on diet quality, we began by identifying the potential economic mechanism(s) by which each program might alter children’s food intake. These included (1) transfer of resources or cash to alter the purchase or use of home-grown foods (hereafter referred to as *resource transfers*); (2) changing food prices to alter purchasing behaviour (hereafter referred to as *access changes*); (3) changing dietary preferences to alter the purchase or use of home-grown foods (hereafter referred to as *preference changes*); and (4) transfer of food items to increase intake (hereafter referred to as *food transfers*). We then used previous studies of each mechanism to quantify the intervention’s likely effect on dietary components involved in five diet–disease relationships for which we had identified evidence for etiologic effects and significant disease burdens in these regions, namely iron and anaemia, vitamin A and mortality, zinc and diarrhoea, zinc and stunting, and animal protein and stunting.

For each program’s impact on any or all four of these dietary components (iron, vitamin A, zinc and animal protein) we then conducted a comprehensive review of the program evaluation literature to identify published studies of similar interventions. This process began with literature searches using the following search terms alone and in combination: impact, diet, diet diversity, iron, zinc, vitamin A, animal protein, fruit, vegetable, dark green leafy vegetable, cash transfer, conditional, poultry production, small livestock production, animal husbandry, home gardens, complementary food production, complementary feeding, mass media campaign, radio campaign, nutrition education, community education, community demonstrations, peer videos, micronutrient sachets, community mills, income elasticity and price elasticity. Those online searches were complemented by direct contacts with the expert participants from our regional meetings.

To identify the most suitable published studies, we searched for outcome and/or impact evaluations that matched the proposed programs on the following criteria: (1) country of interest, (2) target population of interest, (3) mechanism used to alter dietary intake, and (4) target foods and nutrients. In cases where criteria (1) and (2) could not be met, evaluations in other countries and/or target populations in the same region that met the remaining criteria were chosen. Our main countries of interest were India, Ethiopia, and Nigeria, while the larger regions of interest included SSA and South Asia. The target population of interest included children under 5 years of age. Target nutrients of interest included vitamin A, animal protein, iron and zinc. Studies were included if they either reported changes in intakes of these target nutrients or changes in intakes of foods that are major sources of these nutrients.

Studies were excluded if they did not meet any of the aforementioned criteria, if they did not report changes in dietary intake, if they were not experimental in nature, or if they were published before 1995. We also excluded studies from high-income countries [World Bank Classification (The World Bank 2017a)]. In one instance (Educational Entertainment in Ethiopia; see Table 2), the proposed program had only been implemented to change agricultural practices, rather than dietary intake. For this program, we used the existing program’s reported change in uptake of targeted practices as a proxy for changes in dietary behaviours.

From these searches, titles and abstracts were reviewed for relevance using criteria outlined above. The full texts of potentially relevant studies were retrieved. For studies meeting inclusion and exclusion criteria, key data were extracted including country, study design, target population, description of intervention and control groups, intervention components, duration of the intervention, target foods and/or nutrients of intervention, method for assessing dietary intake and intervention effects on diet for the target population. In cases where multiple studies met inclusion criteria for a given program, the closest match was chosen based on our pre-specified criteria outlined above. For each dietary factor of interest, we utilized primary survey data (Global Nutrition and Policy Consortium 2017) to estimate intake by demographic strata within countries (Smith *et al.* 2016). For programs with multiple nutrient targets, multiple impact sources were chosen as necessary to produce impact estimates for all target nutrients. For studies that reported the effects of programs or interventions on food intake rather than nutrient intake, local food composition tables were used to convert food intakes into nutrient intakes.

### Estimating the targeted population for each program

For each of the 12 programs, information on priority target populations was collected at the regional meetings. This information was used in combination with census data or population estimates and demographic data for each country (United Nations 2017) to estimate the total target population for each program. Whenever possible, published reports on potential impact of each program were used to adjust the target population to estimate actual reach, whenever possible. Data on differences between targeted and reached populations were available for three of programs listed in Table 2 from the sources in Table 3; for other programs, costs and impacts were estimated on the basis of reaching the full target population.

**Table 3.** Potential impacts of each program on dietary intake^a^

Country	Program name	Impact mechanism	Impacts on dietary intake per person reached, per day			Sources for behavioural response parameters
			Nutrient targeted	Change in intake (unit/day or %/day)	Disease(s) affected	
Ethiopia	Media and education campaign	Preference change	Iron (mg)	0.98	Anaemia	de Pee *et al.* (1998), Monterrosa *et al.* (2013)
	Educational entertainment	Preference change	Vitamin A (RAE)	36.66	Mortality	Gandhi *et al.* (2009)
	Conditional livestock transfer	Food transfer	Animal protein (g)	2.95	Stunting	Rawlins *et al.* (2014)
			Zinc (mg)	0.30	Stunting, diarrhoea	
	Conditional poultry transfer	Food transfer	Iron (mg)	0.41	Anaemia	Ayele and Peacock (2003)
			Animal protein (g)	2.20	Stunting	
			Zinc (mg)	0.20	Stunting, diarrhoea	
			Iron (mg)	0.30	Anaemia	
	Complementary food production	Food transfer	Zinc (mg)	7.40	Diarrhoea, stunting	Ouedraogo *et al.* (2009)
			Iron (mg)	17.70	Anaemia	
Nigeria	Complementary food processing and sales	Food transfer	Vitamin A (RAE)	26.43	Mortality	Lartey *et al.* (1999)
			Animal protein (g)	20.00	Stunting	
			Zinc (mg)	2.43	Stunting, diarrhoea	
			Iron (mg)	7.21	Anaemia	
	Household animal & horticulture production	Food transfer	Vitamin A (RAE)	335.14	Mortality	Faber *et al.* (2002), Sonaiya (2009)
			Animal protein (g)	1.03	Stunting	
			Zinc (mg)	0.11	Diarrhoea, stunting	
			Iron (mg)	0.15	Anaemia	
	Conditional cash transfer	Resource transfer	Iron (mg)	19%	Anaemia	Ulimwengu *et al.* (2012), Ecker *et al.* (2010)
	Food pricing program	Access change	Vitamin A (RAE)	17	Mortality	Ghana Ministry of Food and Agriculture (2016), USDA (2016)
India	Complementary food processing	Direct transfer	Zinc (mg)	3.34	Stunting, diarrhoea	Hirve *et al.* (2013)
			Iron (mg)	8.14	Anaemia	
	Home gardens	Food transfer	Vitamin A (RAE)	66.50	Mortality	Taher *et al.* (2004), Talukder *et al.* (2010), Chakravarty (2000)
			Animal protein (g)	1.04	Stunting	
			Zinc (mg)	0.22	Stunting, diarrhoea	
			Iron (mg)	1.20	Anaemia	
	Diet diversity media campaign	Preference change	Vitamin A (RAE)	27.95	Vitamin A	de Pee *et al.* (1998), Monterrosa *et al.* (2013)

a Program impacts on dietary intake were estimated from outcome and impact evaluations found through a comprehensive literature search. For programs that targeted multiple nutrients, multiple impact sources were chosen as necessary to produce impact estimates for all target nutrients. For studies that reported the effects of programs/interventions on food intake rather than nutrient intake, local food composition tables were used to convert food intakes into nutrient intakes

### Calibration and validation of program costs

For the 12 selected programs, resources and costs determined from the regional meetings were reviewed for completeness and face validity. Missing or outlier costs were researched in the scientific literature for relevant matches or, if necessary, derived from similar items priced for other interventions within the same region. Costs were distributed across different budget item categories for specificity. Resource needs and costs were calibrated and validated against published reports from similar program interventions identified using the search process described above. Resources and costs were also calibrated and validated across all of the 12 programs so that costs for a given type of resource could easily be compared across the 12 interventions.

Total costs for each program were computed in net present value (NPV) terms to combine start-up and recurring costs, using purchasing power parity (PPP) adjusted prices to facilitate comparisons across countries and over time. PPP adjustment accounts for differences in both currencies and purchasing power in each country. All costs were reported in USD using 2015 PPP exchange rates (The World Bank 2017b). Start-up costs corresponded to the first 12 months of each program, and recurring costs to each subsequent year of intervention. A standard inflation rate of 0.03 per year was applied for costs arising from 2 two through the end of the program, and NPVs were calculated using a discount rate of 0.05.

## Results

### Characteristics of selected programs

The descriptions, target populations and target foods or nutrients for each of the 12 identified programs are detailed in Table 2. In Ethiopia, these included two conditional transfer programs designed to be nutrition-sensitive extensions of the existing Productive Safety Net Program (PSNP), which focused on providing households with either livestock or poultry conditional on household members meeting specific conditions. Two other programs in Ethiopia focused on nutrition education, and one on assisting women to produce complementary infant foods. All five of the Ethiopian programs targeted increased consumption of zinc and iron; four also focused on increasing animal source foods, and one also focused on increasing grains and legumes.

In Nigeria, the programs included a conditional cash transfer program for pregnant women conditional on antenatal care attendance, a food pricing program that taxed sugar-sweetened beverages and subsidized fruits and vegetables, a complementary food program that taught women to produce and sell complementary food, and a program that increased household animal and horticulture production (Table 2). Among these, iron and vitamin A were the most commonly targeted nutrients; two programs were especially comprehensive and targeted iron, vitamin A, zinc and animal protein.

Three priority programs were identified for India, including one focused on complementary infant food processing for low-income families with children, one utilizing a mass media education campaign to increase consumption of vitamin A-rich foods among children under 5, and one establishing home horticulture for rural households with children under 5 (Table 2). Vitamin A, zinc and iron were the most commonly targeted nutrients among these programs, while animal protein would be targeted by one of them.

### Estimated program impacts

The most common identified economic mechanism of impact was direct changes in dietary consumption via food transfers (*N* = 7 programs) (Table 3). Other mechanisms included changes in dietary preferences (*N* = 3), resource transfers for household purchases (*N* = 1) and access improvement (*N* = 1).

Among nutrients targeted, iron was estimated to be the most improved by complementary food production in Ethiopia, with an increase in consumption of 17.7 mg/recipient/day. This program was also estimated to produce the largest increase in zinc intake (7.4 mg/child/day). For vitamin A, the largest estimated increase in intake was associated with the household animal and horticulture production program in Nigeria (335 RAE/child/day); and for animal protein, the largest estimated increase was associated with the complementary food processing program in Nigeria (20.00 g/child/day).

When evaluated by mechanisms of impact, programs involving direct changes in intake via food transfers were generally estimated to produce larger changes in intakes of target nutrients than programs utilizing other mechanisms of impact.

### Estimated program costs

The program costing structures, outlined by budget item, are detailed in Table 4. When comparing individual budget items shared across programs, in Ethiopia the most expensive items were personnel salaries for senior professionals (mean = 45 000 USD/year), skilled personnel (tier 2; mean = 14, 600 USD/year), and professionals (mean = 7800 USD/year). The least expensive items shared across programs included transportation (mean = 0.46 USD/km or 1708 USD/year) and support for volunteers (mean = 140 USD). Budget items that only appeared for one program in Ethiopia, and therefore could not be compared across programs, ranged from 100 000 USD for a consulting contract for radio production and distribution to 12.98 USD/kg of micronutrient powder.

**Table 4.** Price per unit for selected resources used in multiple programs

Country	Item	Unit	Mean^a^ (USD)	Minimum^b^ (USD)	Maximum^c^ (USD)
Ethiopia	Senior professional	Per year	45 000	40 000	60 000
	Professional	Per year	7800	6000	9600
	Skilled personnel—tier 1	Per year	4017	1000	6000
	Skilled personnel—tier 2	Per year	14 600	10 000	18 000
	Unskilled personnel	Per year	4100	1200	7000
	Support for volunteers		140	20	200
	Office space	Per office	2750	500	5000
	Transportation	Per kilometre	0.46	0.28	0.56
	Transportation	Per year	1708	1250	2500
	Micronutrient powder^e^	Per kilogram	12.98	NA	NA
	Annual meeting^e^	Per workshop	50 000	NA	NA
	Mature cow^e^	Purchase value	450	NA	NA
	Radio production and distribution^e^	Consulting contract	100 000	NA	NA
Nigeria	Senior professional	Per year	24 000	18 000	30 000
	Senior administrator—international^e^	Per year	141 176	NA	NA
	Professional (part or full-time)	Per year	7465	2400	18 000
	Skilled personnel	Per year	8300	1500	10 000
	Unskilled personnel^d^	Per year	1000	1000	1000
	Support for volunteers		306	20	800
	Office space	Per year	3000	1000	5000
	Vehicles	Per unit	9093	75	30 000
	Chickens^e^	Per unit	4.6	NA	NA
	Cash transfer^e^	Per recipient	315	NA	NA
	Tree seedlings^e^	Per unit	1.6	NA	NA
	Vegetable seeds^e^	Per unit	1	NA	NA
India	Senior professional	Per year	45 031	40 000	46 729
	Senior administrator—international^e^	Per year	141 176	NA	NA
	Professional (part or full-time)	Per year	11 916	5000	30 000
	Unskilled personnel	Per year	3637	1800	5000
	Office space	Per year	6000	5000	7000
	Micronutrient sachets^e^	Per sachet	0.02	NA	NA
	Small greenhouse^e^	Per unit	150	NA	NA
	Seeds, compost fertilizers, and supplies^e^	Per unit	50	NA	NA
	Cost of airing radio program^e^	Consulting contract	150 000	NA	NA
	Production of television announcements^e^	Consulting contract	200 000	NA	NA
	Food demonstration supplies^e^	Per month	25	NA	NA

*Source*: Costs for each program estimated by workshop participants and project staff were subsequently cross-validated against actual program budgets in the field and against program costing literature

a For items that were only reported once within each country across multiple programs, mean costs are equivalent to the single reported cost. In cases where items were reported multiple times across program budgets within a given country, mean costs are the average cost for that item

b The minimum cost of a single item within each category as specified by workshop participant; reported only if an item appears in multiple program budgets within each country

c The maximum cost of a single item within each category as specified by the workshop participants; reported only if an item appears in multiple program budgets within each country

d Item reported more than once across program budgets, but the cost was the same in each budget for the given country

e This item was only present in one program budget for the given country, and therefore a minimum and maximum cost are not reported; however, items that appeared once were cross-validated with other existing program budgets, costing literature, and expert project staff

For shared budget items across Nigeria programs, the most expensive included senior professionals (mean = 24 000 USD/year), skilled personnel (mean = 8300 USD/year) and vehicles (9093 USD/unit). In comparison, the least expensive included support for volunteers (306 USD), unskilled personnel (mean = 1000 USD/year), and office space (mean = 3000 USD/year). Additional items that were not shared across budgets in Nigeria included chickens, cash transfers, tree seedlings and vegetable seeds.

In India, senior professionals were the most expensive shared budget item (mean = 45 031 USD/year), while unskilled personnel were the least expensive (3637 USD/year). Among items that only appeared in one program budget, the cost of a consulting contract to produce television announcements was most expensive (200 000 USD/contract), while micronutrient sachets were the least (0.02 USD/sachet).

Among the 12 programs, 11 had a specified duration of 5 years, and one had a duration of 3 years (Table 5). Total discounted cost per child reached, shown in Table 5, ranged from USD 2650 for a livestock transfer program to 0.58 for a media and education campaign, both in Ethiopia. In other countries total cost per child ranged from USD 1919 for a cash transfer program to 2.62 for a food pricing program in Nigeria, and from USD 586 for home gardens to 27 for a media campaign in India. The most expensive programs per child used transfers of valuable assets such as livestock, garden supplies and cash, while the least costly programs used outreach and food pricing or market access such as for complementary foods in Ethiopia.

**Table 5.** Duration, size of target population and total costs per child targeted by each program^a^

Country	Program name	Length of program (years)	Number of children targeted^b^	Start-up cost per child targetedd (USD)	Recurring cost per child targetedee (USD/year)	Discounted NPV per child targeted (USD)^f^
Ethiopia	Conditional livestock transfer	5	941 200	522	552	2650
	Conditional poultry transfer	5	1 568 600^c^	141	147	709
	Media & education campaign	3	7 848 700^c^	0.2	0.2	0.6
	Educational entertainment	5	14 600 000	6.48	5.59	28
	Complementary food production	5	1 449 000	1.8	1.9	9.1
Nigeria	Conditional cash transfer	5	21 953 300	380	399	1919
	Food pricing program	5	18 043 200^c^	0.92	0.95	2.62
	Complementary food processing and sales	5	360 000	34	35	169
	Household animal & horticulture production	5	6 500 000	203	214	1026
India	Complementary food processing	5	114 123 000	7.75	7.67	37
	Diet diversity media campaign	5	129 600	9.06	4.7	27
	Home gardens	5	83 95 600	118	121	586

NPV, net present value; SSB, sugar sweetened beverage

a All past values are adjusted to USD using 2015 PPP exchange rates for each year from World Bank, World Development Indicators

b The size of the targeted population was calculated using information on target populations that was collected in program descriptions obtained at the regional meetings in combination with census data or population estimates and demographic data for each country

c Sources that were used to determine the impact of each program on dietary intake were also used to estimate the size of the reached population, when possible, based on estimates of program coverage or uptake. For example, if an impact source estimated program coverage to be 50%, the target population was adjusted accordingly to produce an estimate of the reached population. In cases where estimates of program reach were not available, the target population was equal to the reached population

d Refers to all costs incurred in the first 12 months of the program

e All costs pertaining to the program after its first year of implementation; an inflation rate of 0.05 applies to every year of the program beyond the ‘start-up’ year until the program’s conclusion

f Sum of start-up and recurring costs over the length of the program using an inflation rate of 0.05 and a discount rate of 0.03 over the duration of the program. This is not an estimate of cost-effectiveness and should be considered in the context of health benefits along with program specific measures

## Discussion

### Main findings

This study provides novel estimates of estimated budgetary costs and potential impact on child dietary quality of 12 nutrition-sensitive interventions in Ethiopia, Nigeria and India, using a mixed methods participatory approach including regional stakeholders from diverse sectoral, institutional and disciplinary backgrounds to identify programs of interest with their key components and mechanisms, followed by literature reviews to produce a calibrated and validated set of budgets and impact estimates. This methodology offers a promising approach to estimating the costs and dietary impacts of nutrition-sensitive programs in resource-limited settings.

A principal finding is that stakeholder-designed interventions achieved the largest potential changes in child nutrient intake via food and resource transfers, rather than via market prices or other mechanisms. Such programs included transfers of poultry and other livestock, assistance with complementary food production, and resources necessary for homestead gardens. This finding is consistent with prior literature highlighting the benefits of similar programs on dietary quality. For example, livestock production programs have been found to improve dietary intakes among the poor by providing a regular supply of animal–source foods that are rich in nutrients such as zinc, iron and animal protein, with less susceptibility to seasonal fluctuations (Randolph *et al.* 2007). In addition, home gardens and homestead food production programs have been found to improve maternal and child intakes of target foods and nutrients and to increase dietary diversity (Ruel and Alderman 2013, Webb and Kennedy 2014). Finally, a systematic review of complementary feeding interventions found that those involving education alone for mothers on appropriate complementary feeding have a modest impact on recommended micronutrient intakes, while fortification strategies for complementary foods such as prioritized in our programs have a larger impact on micronutrient intakes (Dewey and Adu-Afarwuah 2008). Our results align with these findings by showing a larger impact on iron and zinc intakes of the proposed complementary food fortification and production program in Ethiopia when compared with the complementary food processing program in Nigeria that focused on education but not direct fortification.

A second important finding is that food and resource transfers are the costliest programs per child targeted. Programs that aimed to alter preferences, change market prices or otherwise improve access to healthy foods tended to be less costly per child, even though some of these achieved comparable estimated levels of changes in dietary intake. These findings highlight the need for future formal cost-effectiveness analyses and comparisons of these very different programs for each target population. Indeed, our results provide a foundation of methods, costs, and impacts for the development of appropriate modelling approaches, parameters and sensitivity analyses to assess cost-effectiveness. For instance, it may be that beneficiaries in more remote areas are best reached via transfers, while households closer to markets may be reached more cost-effectively via programs to alter prices and promote behaviour change. The overall cost-effectiveness of either kind of program will also depend on the numbers of beneficiaries and their relative risks for various disease outcomes associated with changes in dietary intake.

### Strengths

An important feature of this analysis is that interventions considered were selected and defined through a participatory process including a diverse group of regional experts in SSA and South Asia. These stakeholder consultations ensured that the interventions described in the study incorporated local knowledge and expertise from a range of sectoral, disciplinary and institutional backgrounds, which also helps ensure accuracy and relevance for policymaking in each country setting (Victora *et al.* 2012; Holdsworth *et al.* 2015). Importantly, these methods also limited the opportunity for any single interested party to influence results in their favour, a challenge for prior program evaluations often performed by the implementing agency, funding sponsor or other interested party (Every-Palmer and Howick 2014). We identified and focused upon specific diet–disease relationships with evidence for etiologic effects and relevant burdens for maternal-child health in these regions. A mixed methods approach allowed us to incorporate calibration and validation of program resources, costs, and impacts based on existing evidence.

### Limitations

While our mixed methods approach and stakeholder engagement increase the potential relevance of the results to local decision-making, such methods preclude comprehensive assessment of every possible program iteration. The data presented here should be considered central estimates for costs and impacts of 12 specific programs for these countries. Future analyses should formally consider scientific and sampling uncertainty, for example incorporated as part of sensitivity analyses in subsequent cost-effectiveness analyses. Our methods focused on SSA and South Asia, and subsequently on Ethiopia, Nigeria and India as major representative countries; and our findings may be less generalizable to other countries or regions. On the other hand, the approach described here provides a roadmap for similar assessments of nutrition-sensitive interventions to improve diet quality in other countries.

## Conclusions

We identified and characterized 12 specific programs to improve diet quality and child health in Ethiopia, Nigeria and India, along with estimated resource costs and dietary impacts. These methods and results can help address crucial knowledge gaps relating to nutrition-sensitive interventions targeting maternal-child health in low- and middle-income countries. The findings may inform ongoing policy discussions to meet national and international nutrition goals, and can also serve as critical inputs to future cost-effectiveness analyses of programs to improve the well-being of children in resource-limited settings.
